# CanISO: a database of genomic and transcriptomic variations in domestic dog (*Canis lupus familiaris*)

**DOI:** 10.1186/s12864-023-09655-0

**Published:** 2023-10-13

**Authors:** In Seok Yang, Insu Jang, Jin Ok Yang, Jinhyuk Choi, Min-Seo Kim, Ka-Kyung Kim, Byung-Joon Seung, Jae-Ho Cheong, Jung-Hyang Sur, Hojung Nam, Byungwook Lee, Junho Kim, Sangwoo Kim

**Affiliations:** 1 Department of Biomedical Systems Informatics, Yonsei University College of Medicine, Seoul, 03722 Korea; 2 Korea Bioinformation Center (KOBIC), Korea Research Institute of Bioscience & Biotechnology, Daejeon, 34141 Korea; 3 Department of Veterinary Pathology, College of Veterinary Medicine, Konkuk University, Seoul, 05029 Korea; 4 School of Electrical Engineering and Computer Science, Gwangju Institute of Science and Technology (GIST), Gwangju, 61005 Korea; 5 Department of Biological Sciences, Sungkyunkwan University, Suwon, 16419 Korea

**Keywords:** Domestic dog, Canine mammary tumor, SNP, Transcriptome, Transcript isoform, Database

## Abstract

**Background:**

The domestic dog, *Canis lupus familiaris*, is a companion animal for humans as well as an animal model in cancer research due to similar spontaneous occurrence of cancers as humans. Despite the social and biological importance of dogs, the catalogue of genomic variations and transcripts for dogs is relatively incomplete.

**Results:**

We developed CanISO, a new database to hold a large collection of transcriptome profiles and genomic variations for domestic dogs. CanISO provides 87,692 novel transcript isoforms and 60,992 known isoforms from whole transcriptome sequencing of canine tumors (*N* = 157) and their matched normal tissues (*N* = 64). CanISO also provides genomic variation information for 210,444 unique germline single nucleotide polymorphisms (SNPs) from the whole exome sequencing of 183 dogs, with a query system that searches gene- and transcript-level information as well as covered SNPs. Transcriptome profiles can be compared with corresponding human transcript isoforms at a tissue level, or between sample groups to identify tumor-specific gene expression and alternative splicing patterns.

**Conclusions:**

CanISO is expected to increase understanding of the dog genome and transcriptome, as well as its functional associations with humans, such as shared/distinct mechanisms of cancer. CanISO is publicly available at https://www.kobic.re.kr/caniso/.

**Supplementary Information:**

The online version contains supplementary material available at 10.1186/s12864-023-09655-0.

## Background

The domestic dog, *Canis lupus familiaris*, is one of the most influential animals to humans in terms of social, cultural, and biological relationships. Dogs have been living with humans for thousands of years [1, 2] since branching off from wolves about 15,000 − 100,000 years ago [3], and are now regarded as companion animals and great assistants to humans. Due to their unusual evolutionary process, wherein a strong selective pressure forced rapid diversification and exceptional intimacy in a relatively short time, studies of the relevant genomic and transcriptomic profiling are being conducted [1, 4–7]. Moreover, understanding the molecular mechanisms of diseases in dogs has drawn interest in recent years, especially for cancer [8, 9]. Dogs generally share a living environment with humans and both acquire spontaneously occurring cancers, making dogs the closest animal cancer model to humans [10–12]. Researchers expect that comparative analysis of the genome and transcriptome of diseases in dogs will lead to a better understanding of therapeutic strategies for diseases in humans [13], while the longevity of dogs is also a valuable topic to study [14].

Despite the importance, a lack of in-depth genomic and transcriptomic information in dogs has limited the range of genomic studies. For example, while dogs have a similar number of protein coding genes (*N* = 20,257) as mice (*N* = 22,508), the number of genomic short variants in dogs (approximately 6 million) has been catalogued only about 7% of that in mice so far (approximately 84 million); this is less than 1% of the number in humans (approximately 666 million, Additional file 2: Table S1) [15]. Likewise, in transcriptomic profiles, far smaller number of transcript isoforms have been identified (60,994 in dogs, compared to 227,530 and 142,446 in humans and mice) [15]. Although recent efforts on constructing large-scale databases such as iDog [16] have been alleviating the shortage of the information, more active generation of high-throughput sequencing data on individual dogs and their use for extracting and updating genomic and transcriptomic information is urgently demanded.

Here we developed a new database CanISO that provides information for 210,444 unique germline SNPs as genomic variations (122,900 known and 87,544 unreported SNPs) and 87,692 novel transcript isoforms (35,062 and 69,337 in normal and tumor conditions, respectively) together with 60,992 known isoforms as transcriptome data. Compared to iDog database, CanISO has strengths in providing detailed information on transcript isoforms (e.g., annotation and expression levels). These data were coupled with a convenient web-based query system to search and compare SNP and transcriptome profiles, facilitating researcher-driven comparative analysis. We anticipated that CanISO will be a useful repository for researchers in veterinary science, comparative genomics, and even human cancer genomics.

## Construction and contents

### Data generation and processing

#### Sample collection and sequencing

In this study, we included fresh and formalin-fixed paraffin-embedded tumor tissues (*N* = 183) of canine mammary tumors (CMTs) and their matched adjacent normal mammary tissues (*N* = 64) that were reported in our previous study [8]. The detailed protocols for sample collection and sequencing are described as follows. Whole transcriptome sequencing (RNA-seq) was conducted for 157 CMTs and 64 matched normal mammary tissues. Whole exome sequencing (WES) was performed on the blood of 183 dogs. Both RNA-seq and WES were conducted with Illumina HiSeq 2500 and its designated protocols.

#### Identification of transcript isoforms

RNA-seq data consisting of 157 tumor samples and 64 normal samples matched with tumors were used (SRA accession ID: SRP159466 [17]). An iterative RNA-seq analysis pipeline was employed to maximize the discovery of novel transcript isoforms (Additional file 1: Fig. S1a), which was composed of six steps: (i) read alignment, (ii) transcript reconstruction, (iii) initial (1^st^) filtration of isoforms, (iv) expression quantification, and (v and vi) two additional (2^nd^ and 3^rd^) filtration processes of isoforms.

Raw sequence reads (FASTQ) were aligned to the canine reference genome CanFam3.1 using TopHat2 (version 2.0.12 [18]) with Ensembl gene annotation (Release 98 [15]) (step i). Transcriptome reconstruction was performed using StringTie (version 2.0.6 [19]) and then merged into a gene transfer format (GTF) file with the merge function of the program (step ii). The 1^st^ filtering step was done to remove the isoforms without strand information from the GTF file (step iii). Expression levels in transcripts per million (TPM) of transcript isoforms in each sample were quantified by using RSEM (version 1.3.0 [20]) (step iv). The 2^nd^ filtering step was performed to filter out novel transcript isoforms that were expressed in < 10 samples (Additional file 1: Fig. S2a), those that were mapped in intergenic regions (unmapped) or poorly mapped (< 30%) in a gene region, or those identified as a fused isoform of two gene products (Additional file 1: Fig. S2b) (step v). The 3^rd^ filtering step was applied to filter out the remaining isoforms with any exon that was covered < 90% by sequence reads (Additional file 1: Fig. S2c) and those that contained no novel junction or any junctions (i.e., introns) at which < 10 spliced reads were passed (Additional file 1: Fig. S2d) (step vi). During the 2^nd^ and 3^rd^ filtering steps, three confidence values were added to the annotations of the remaining novel isoforms based on the respective thresholds.

The entire process was repeated until newly discovered transcript isoforms at each cycle over their cumulative counts were < 1%. Through the cyclic process, an output GTF file was obtained in each condition. All novel transcript isoforms were then compared between the two conditions by considering the positions of exon boundaries except for the transcription start and termination sites of the isoforms. If a novel isoform in the tumor condition matched an isoform in the normal condition, it was considered as a normal one and its annotation was replaced from the tumor GTF file.

Using finally obtained GTF files, the expression levels of the transcript isoforms in each condition were quantified. In addition, the gene- and isoform-wise expression data in each condition were processed to obtain the statistical parameters (lower outliers, minimum, first quartile, median, third quartile, maximum, and upper outliers), which were visualized to compare between normal and tumor conditions.

#### Determination of major-isoform-switched genes

For the genes with ≥ 2 transcript isoforms, the aim was to identify major-isoform-switched genes according to the condition change from normal to tumor. Note that a major isoform was defined as the most expressed isoform in a given gene. At first, the major isoforms were determined based on the median expression levels for all samples in each condition. If the median expression level was zero for a certain isoform in a condition, the average value was used instead of the median value. Their switching from normal to tumor conditions was then investigated in each gene.

#### Investigation of alternative splicing patterns among transcript isoforms

Alternative splicing patterns—alternative promoters (APR), cassette exons (CE), cryptic exons (CRE), mutually exclusive exons (ME), alternative 5' (A5SS) and 3' splice sites (A3SS), retained introns (RI), cryptic introns (CRI), and alternative polyadenylation (APA)—were investigated between transcript isoforms (Additional file 1: Fig. S3). Annotations of transcript isoforms were used in the GTF files obtained from normal and tumor conditions. To determine alternative splicing patterns in the genes with ≥ 2 transcript isoforms, canonical isoforms were used as references, which were obtained from Ensembl genome browser [15]. If the canonical isoform did not exist in a gene, the longest transcript isoform and/or with an entry ID of UniProt [21] was selected as an alternative. Then, alternative splicing patterns were counted by comparing the exon and intron structures in each isoform pair using the in-house script. In this step, only the isoforms that shared the regions with the reference isoform for a given gene were considered.

#### Generation of nucleotide and amino-acid sequences from transcript isoforms

For novel transcript isoforms, the nucleotide sequences were first determined based on their annotation, and the sequences were then divided into two groups according to the presence or absence of known translation start sites. If the site existed in an isoform, it was used to deduce amino acid sequences of the isoform. Otherwise, the longest amino acid sequence that can be made for each transcript isoform was selected by considering three open reading frames according to the strand direction under the ATG-dependent translation rule using the in-house script. For known transcript isoforms, nucleotide and amino acid sequences were downloaded from Ensembl (Release 98 [15]).

#### Determination of translation states of transcript isoforms

The translation states of novel transcript isoforms were predicted by considering that the whole region of the first and last exons could be 5' and 3' untranslated regions, respectively. Translation states were defined as follows: 1) A “fully translated isoform” was defined as the isoforms that were translated from the first or second exons to the last or second last ones. 2) A “translation truncated isoform” was defined as the isoform where translation started from the first or second exons and unusual termination was found in an exon that was not the last or second last one. 3) If an amino acid sequence of an isoform was determined but translation start site did not exist in the first and second exons, it was considered an “unusually translated isoform.” 4) If no amino acid sequence for an isoform was determined, it was considered to be a “non-coding isoform.”

#### Inference of tumor-specific isoforms

Tumor-specific isoforms were inferred by applying the definitions (type I and II) used in our previous study [22]. Briefly, type I indicates tumor isoforms accompanying the change of expression status (unexpressed to expressed) according to the condition change (normal to tumor), where the expression status was determined with a minimum threshold of median TPM > 10^−6^. Type II represents major isoforms of major-isoform-switched genes in the tumor condition with a sufficient change (median TPM fold change ≥ 2) in expression level. The type I and II tumor isoforms with the thresholds of expression level (≥ 1.0 median TPM), occupancy (≥ 10%), and translation status (fully translated or predicted so) were filtered to select strong candidates (level 1). The tumor isoforms that were also expressed above the thresholds were further filtered out for expression and occupancy in the normal condition (level 2). Finally, a stringent approach was taken to obtain cancer-related transcript isoforms by examining the existence of their genes that were matched to the human cancer census genes (*N* = 723; Census_allThu_Aug_8_2019.csv from the COSMIC database [23]).

#### Identification of genomic variants in dog

Germline SNPs were identified by analyzing the WES data (SRA accession ID: SRP159481 [17]) as shown in Additional file 1: Fig. S1b. Raw sequencing reads (FASTQ) were aligned to the CanFam3.1 reference genome using BWA-MEM2 (version 0.7.17 [24]) to acquire read alignment (BAM). Pileup files were generated using VCFtools (version 0.1.12b [25]). Germline SNPs were called using VarScan2 (version 2.4.3 [26]) with options of “–min-coverage 10” and “–min-avg-qual 20” from pileup files generated using SAMtools (version 1.9 [27]). Filtration was conducted on the called SNPs by the indicated target regions using VCFtools (version 0.1.12b [25]). Functional effects of the passed SNPs were annotated using Ensembl Variant Effect Predictor (VEP) [28]. Final variant sets were written in variant call format (VCF) and used for further analysis.

#### SNP-based sequence generation to infer dog breeds

Reference SNP data (*N* = 150,131) were obtained for 164 pure dog breeds from a previous study [1]. SNP sequences were then produced by extracting the genomic positions of SNPs matched between pure breeds and our samples, sorting the positions in order of chromosome number and nucleotide position, and then concatenating SNP alleles. In this process, each nucleotide in the sequences was determined according to the zygosity of each SNP allele (i.e., homozygous or heterozygous). For example, when there were two alleles (“G” and “C” as reference and alternative alleles, respectively) at a SNP position, if the SNP allele was homozygous (“G/G” or “C/C”), its nucleotide was denoted as the corresponding allele in uppercase (i.e., “G” or “C”). Otherwise (i.e., heterozygous; “G/C”), the alternative lowercase allele (i.e., “c”) became its nucleotide. The SNP sequences were utilized to infer the dog breeds of our samples by comparing sequences between pure breeds and our samples. Since there were ≥ 2 SNP data for each pure breed, maximum sequence identity was used as a metric to determine the best matched pure breeds.

#### Processing of gene/isoform information of dog and human

To provide basic information of the genes and transcript isoforms and to utilize it in the search function, files containing gene/isoform information of dogs (*C. lupus familiaris*; NCBI tax_id: 9615) and humans (*Homo sapiens*, NCBI tax_id: 9606) were downloaded from Ensembl (Release 98 [15]) and NCBI Gene (gene_info and gene2ensembl; updated on 17 Dec 2019 [29]), respectively. Finally, the gene/isoform information from Ensembl and NCBI Gene was merged by linking gene_id and transcript_id between the downloaded files.

#### Processing of human gene expression data

To obtain the gene expression data of human breast tissues used for the comparison with dog mammary tissues, the data were downloaded from GTEx Portal (Gene TPMs and Transcript TPMs, version 7) [30]. After extracting information from human breast tissue, the data were further processed to obtain quartiles for the expression levels of each gene and its isoforms.

#### Comparison of transcript isoform sequences between dog and human

We first determined common genes between dog and human by matching their gene symbols (*N* = 16,375; Additional file 1: Fig. S9). Then, we prepared nucleotide sequences of dog isoforms as described earlier, and obtained human isoforms by indexing reference genome sequence (Homo_sapiens_assembly19.fasta.gz) using RSEM [20] with annotation (gencode.v19.transcripts.patched_contigs.gtf) from GTEx portal [30]. Then, we calculated normalized Google distances between two isoform sequences of dog and human using a python-based tool, Alfree [31]. If the distance for an isoform pair was less than or equal to 0.1, we considered the pair showing high similarity between the two species.

#### Identification of transcriptome similarity between dog and human

To identify transcriptome similarity between dog and human, we also used the common genes determined above (*N* = 16,375; Additional file 1: Fig. S9). Dog gene expression levels in TPM were estimated using RSEM (version 1.3.0 [20]). Human gene expression data in TPM were downloaded for 30 normal tissues from GTEx Portal [30] and 32 cancer types from NCI GDC Data Portal [32]. After obtaining median gene expression levels for the genes in each tissue, we calculated Pearson and Spearman coefficients to show gene expression similarities in tumor and even normal conditions between dog and human.

#### Scoring key signature pathways

We employed Altered Pathway Analyzer (version 3 [33]) to determine scores of key signature pathways associated with tumorigenesis. We then prepared input files of the tool as follows. First, we generated read count matrices in normal and tumor conditions as case and control expression profiles from BAM files for CMT samples using SAMtools (version 1.9 [27]) and HTSeq (version 0.12.4 [34]). In this process, gene IDs or names were converted to entrez IDs that are used in NCBI Gene database [29]. Second, we downloaded read count files in normal and cancer conditions from NCI GDC Data Portal [32] for human breast cancer type (BRCA) to compare with the CMT samples. Third, we prepared a pathway set containing 214 KEGG pathways [35] by adding several signaling and metabolic pathways that are missed in default pathway set; and removing disease pathways that cover multiple pathways, making it difficult to detect key pathways.

#### Database construction and web deployment

Essential data were prepared as follows: i) annotations, expression data, and sequences (nucleotide and amino acid) of genes and transcript isoforms in normal and tumor conditions for dog mammary tissues; ii) dog germline SNPs obtained from the normal condition; iii) expression data for human normal breast tissues; and iv) merged gene/isoform information. The data were stored in a relational DB (MySQL 5.6). Apache (version 2.2) and Tomcat (version 7.0) were employed for web deployment. JAVA, JavaScript, and Highcharts were used to implement the CanISO user interface.

### Data integrated in CanISO

**Fig. 1:**
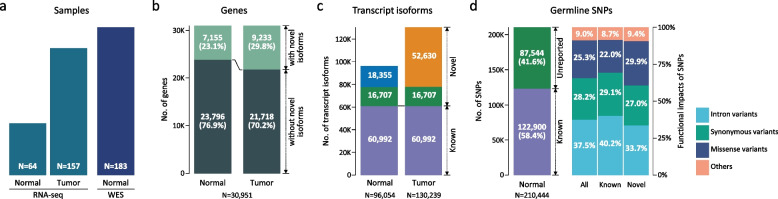
Summary of data included in the CanISO database. **a** Number of samples in RNA-seq (left) and WES datasets (right). **b** Gene counts with or without novel transcript isoforms in normal and tumor conditions. **c** Number of transcript isoforms in normal and tumor conditions. Blue and orange indicate novel isoforms specific to each condition. Green indicates shared novel isoforms in both conditions. Purple represents known isoforms. **d** Number of germline SNPs included in the database. Proportion of unreported SNPs (left) and different functional groups (right) are depicted. Functional impacts were predicted by VEP

Figure 1a provides a summary of RNA-seq (left) and WES datasets (right) integrated in CanISO, which were derived from normal and tumor samples of domestic dogs with CMT (*N* = 183). CanISO mainly consists of transcriptome and germline SNP information obtained by analyzing the RNA-seq and WES datasets, respectively.

#### Transcriptome data

The overall pipeline for identifying transcript isoforms from RNA-seq samples of normal (*N* = 64) and tumor (*N* = 157) tissues is depicted in Fig. S1a in Additional file 1. The pipeline produces two major sets of information: measured gene expression levels and transcript profiles including novel isoforms. For the calculation of gene expression, TopHat2 [18] and StringTie [19] were used with the raw sequencing file (FASTQ) and reference genome (CanFam 3.1.98) (see Methods for detail). For the maximized discovery of novel transcripts, an iterative procedure was exploited (Additional file 1: Fig. S1a). For each iteration, transcripts that were not matched to previously known isoforms (CanFam3.1.98 Ensembl annotation [15]) were further filtered out with stringent criteria, such as expression level and a number of samples, to secure confidence (see Methods for detail). After repeated discovery cycles on 60,992 known reference transcripts, a total of 96,054 and 130,239 transcript isoforms were identified from normal and tumor samples, respectively (Additional file 2: Table S2). Novel isoforms accounted for 53.2% (69,337) and 36.5% (35,062) of total transcripts in tumor and normal samples, suggesting that current knowledge about the canine transcriptome is still lacking in tumor and even normal conditions.

Of a total of 30,951 genes, 7,155 (23.1%) and 9,233 genes (29.8%) were found to have at least one novel isoform in normal and tumor conditions, respectively (Fig. 1b). Because novel isoforms were independently discovered in each condition, annotations of novel isoforms were compared between the two conditions. Overall, 18,355 and 52,630 novel isoforms were observed to be appeared only in normal and tumor conditions, and 16,707 novel isoforms were found to be shared in both conditions (Fig. 1c).

Annotations in GTF were prepared for total transcript isoforms—96,054 and 130,329 transcript isoforms in normal and tumor conditions—and expression files in comma-separated values format were then generated by gene- and isoform-level quantification in TPM with these annotations. When median expression levels of all transcript isoforms were examined in each condition, 26,160 (27.2%) and 29,819 (22.9%) isoforms were generally expressed (≥ 1 TPM) in normal and tumor conditions, among which 12,430 (47.5%) and 17,104 (57.5%) were novel transcript isoforms. Nucleotide and amino acid sequence files were also prepared in FASTA format for known and novel transcript isoforms in each condition. For novel ones, amino acid sequences were generated based on known translation start sites or the longest sequences that were predicted in given nucleotide sequences. Finally, transcript annotations, expression profiles, and nucleotide/amino acid sequences were systemically stored in the CanISO database for web service and data release.

#### Germline SNP data

WES data analysis pipeline was constructed to identify germline SNPs (Additional file 1: Fig. S1b). A total of 210,444 unique on-target SNPs was identified from WES datasets for 183 normal dog samples (50,605 SNPs on average). Among them, a total of 87,544 SNPs (41.6%) were found to be novel, which have not been previously reported in NCBI dbSNP, demonstrating again a lack of information on the canine genome. VCF files containing the on-target SNPs per sample and of all samples were prepared for data release.

Functional impact of the SNPs was also examined (Fig. 1d); three terms (intron_variant, synonymous_variant, and missense_variant) were most frequently observed, and higher proportion of missense variants was observed in novel SNPs (28,357; 29.9%) than known ones (27,358; 22.0%). Only a few SNPs (1,491; 0.8%) were associated with the following five terms (splice_acceptor_variant, splice_donor_variant, stop_gained, stop_lost, or start_lost) whose functional impact would be high.

### Implementation of the CanISO database and web pages

**Fig. 2:**
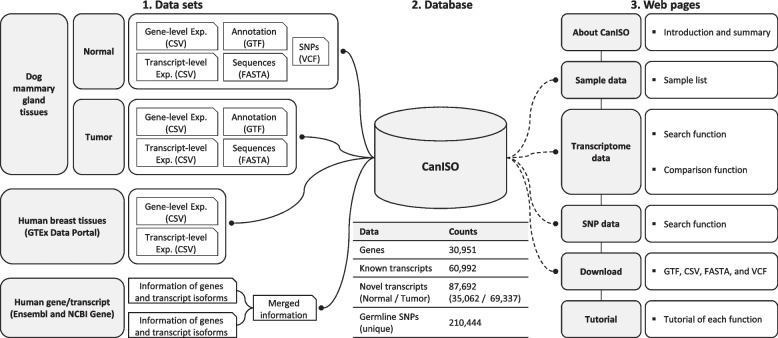
Schematic relationship between datasets, database, and web pages. (Left) Three different datasets used to construct the integrated database: 1) data obtained by analyzing RNA-seq and WES datasets of dog samples, 2) public gene expression data of human breast tissues, and 3) human gene/isoform information. (Middle) Data statistics of genes, transcripts, and germline SNPs in the CanISO database. (Right) Configuration of the CanISO web pages

As shown in Fig. 2, the CanISO database contains five major types of information derived from RNA-seq and WES datasets: 1) gene-level expression, 2) transcript-level expression, 3) gene and transcript annotations, 4) transcript-based nucleotide and amino acid sequences, and 5) germline SNPs. For comparative analysis with human data, expression data of human breast tissue from the GTEx Portal [30] and gene/isoform information from Ensembl [15] and NCBI Gene [29] databases were also incorporated. Web pages were constructed on top of the integrated database for querying and visualizing genomic and transcriptomic profiles and for public distribution of the data.

**Fig. 3:**
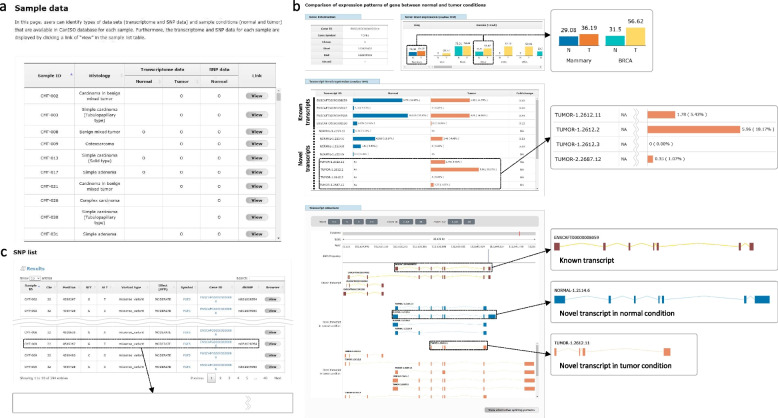
Data visualization of the CanISO web pages. (**a**) Table showing the presence or absence of the transcriptome and/or SNP data for each sample. An open circle indicates the existence of the corresponding data. Users can move to the detailed sample data page for a selected sample by clicking the “view” button. (**b**) Comparison of gene/transcript-level expression (top and middle) and transcript structures (bottom) of the *TGFB1* gene between normal and tumor conditions. In the middle figure, NA means “not applicable” due to the absence of the corresponding isoforms in each condition. (**c**) Search results of germline SNPs for the *FGF5* gene. Users can click the “view” button to view the genome browser page showing the frequencies of the selected and neighboring SNPs

CanISO consists of three main web pages for sample, histology, transcriptome, and SNP information. First, the sample data page allows users to check which sample contains the transcriptome and/or SNP data according to the sample conditions (Fig. 3a), where users can view the transcriptome and SNP data for a selected sample (Additional file 1: Fig. S4).

Second, the transcriptome data page supports search and comparison functions. The search function is based on a query system oriented to genes of interest for each sample condition (normal or tumor). The comparison function allows users to compare transcriptome data of normal and tumor conditions for domestic dogs. For example, Fig. 3b shows the comparison results of gene/transcript-level expression and transcript structures between both conditions for *TGFB1*, respectively, where users can observe not only gene-level comparison results between CMT samples and 32 human cancer types (the top figure), but also the occurrence of novel isoforms in tumor condition (enlarged image in the middle figure) with their detailed structures (enlarged images in the bottom figure), which is useful information since CMT is known as a great model for human breast cancer [7, 8, 12]. Furthermore, the function provides comparison results of transcriptome data between dog mammary and 30 human tissues in normal condition (Additional file 1: Fig. S5).

Third, the SNP data page supports a search function to retrieve germline SNPs. Figure 3c shows an SNP search result for the *FGF5* gene, of which alleles were associated with seven different coat phenotypes in dogs [36]. Users can find detailed SNP information, including genomic location, variant type, and functional annotation with predicted effect (enlarged image for a SNP).

### Utility and Discussion

As mentioned above, CanISO provides a query system that allows easy searching and comparing transcriptome and germline SNP profiles on the web, and also supports a direct download function of bulk data from the integrated database. Thus, it is expected that CanISO may facilitate the usability of the data for various research purposes. Here, we demonstrate some advanced analyses using the data as potential applications of the CanISO database.

#### Inference of dog breeds using germline SNPs

To date, genome-wide SNP and haplotype analyses have been conducted to identify evolutionary relationships between domestic dogs and grey wolves and to infer the geographic origin or ancestry of the dogs [1, 4, 5]. These efforts have made it possible to identify dog breeds using SNP information. Thus, we implemented a module to infer dog breeds with germline SNP data and tested it. From our samples, a total of 2,922 SNP positions were matched with those of pure dog breeds, and thus SNP sequences that consist of 2,922 nucleotides for 164 pure breeds and our 183 dog samples were generated.

**Fig. 4:**
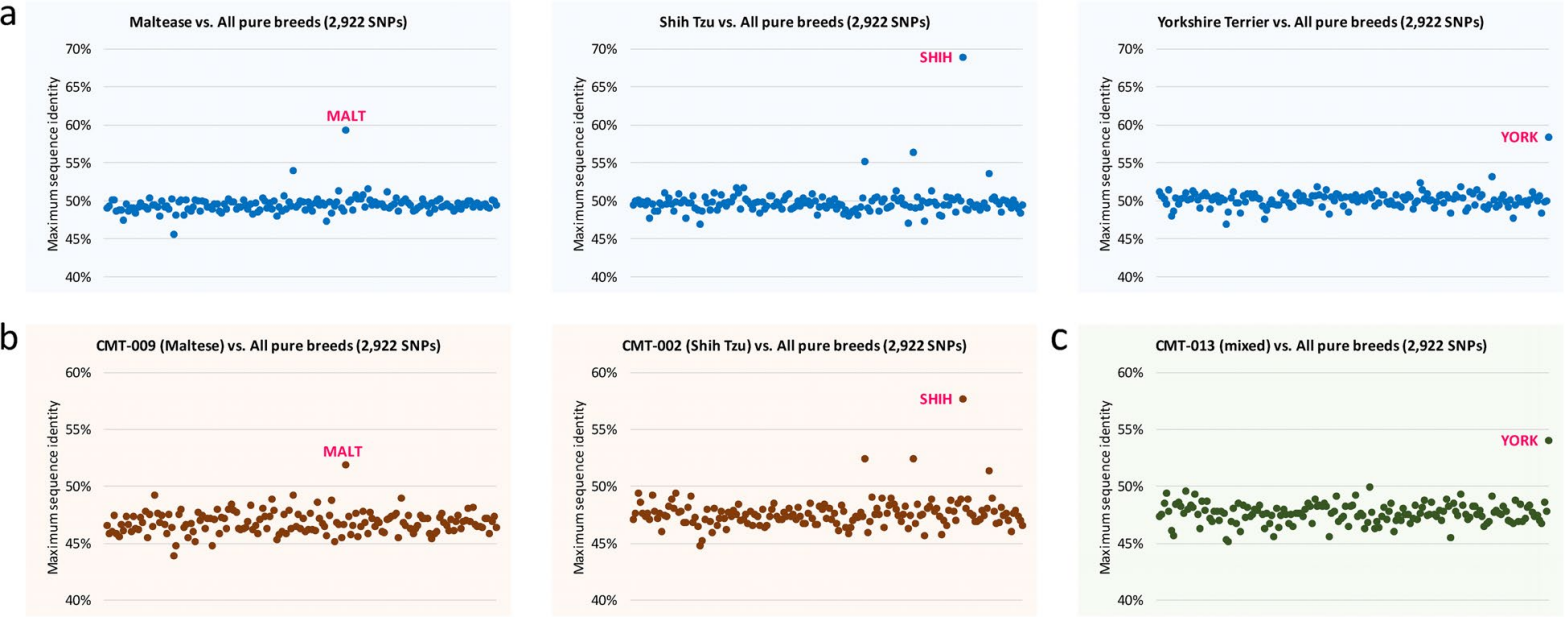
Results of dog breed inference using sequences generated with 2,922 SNPs. (**a**) Inference results of three pure breeds (MALT: Maltese; SHIH: Shih Tzu; and YORK: Yorkshire Terrier). (**b**) Inference results of two dog samples (CMT-009: Maltese and CMT-002: Shih Tzu). (**c**) Inference result of a dog sample that was recorded as “mixed” by the dog owner. The dog was predicted to be closest to a Yorkshire Terrier

At first, the accuracy of dog breed inference was investigated through comparison using only pure dog breed sequences. The module accurately identified the breeds (Fig. 4a), in which Maltese, Shih Tzu, and Yorkshire Terrier exhibited maximum sequence identities of 59.1%, 64.1%, and 58.4%, respectively, compared to other breeds. Although the use of a small set of SNPs (*N* = 2,922) resulted in a loss of informative SNPs discriminating dog breeds—thereby leading to relatively low sequence identities (i.e., low-resolution power) when compared with the results using 150,131 SNPs (~ 100%, Additional file 1: Fig. S6)—the similarity values clearly identified the true breeds. Then, the same approach was tested with SNP sequences of our samples, and it was found that the best-matched pure breeds were consistent with the breed names of our samples that were recorded by dog owners with high accuracy (92.9%; 144 of 155 dogs), supporting reliable performance for dog breed inference (Fig. 4b). This approach can be directly used to infer the breed of unknown samples. We tested it with one sample recorded as “mixed”, and found that the sample was closest to a Yorkshire Terrier with significant similarity differences compared to other breeds (Fig. 4c), enabling the imputation of missing information.

Consequently, the dog breeds of our samples were successfully inferred with high accuracy by employing a similarity search with SNP sequences. We expect that advanced application of a well-fitted probability model would provide more accurate inference results for the closest breeds.

#### Utilization of isoform-centric analysis

Advances in bioinformatics algorithms and pipelines have made it possible to dissect human gene expression at the isoform level and have revealed unique features related to diseases such as cancer [37, 38]. However, dog transcriptome has not been explored as deeply as in humans. Here, isoform-centric analyses of canine tumor data using CanISO database are presented, showing interesting results that may underlie tumor progression.

##### Identification of abnormal splicing patterns in tumor condition

**Fig. 5:**
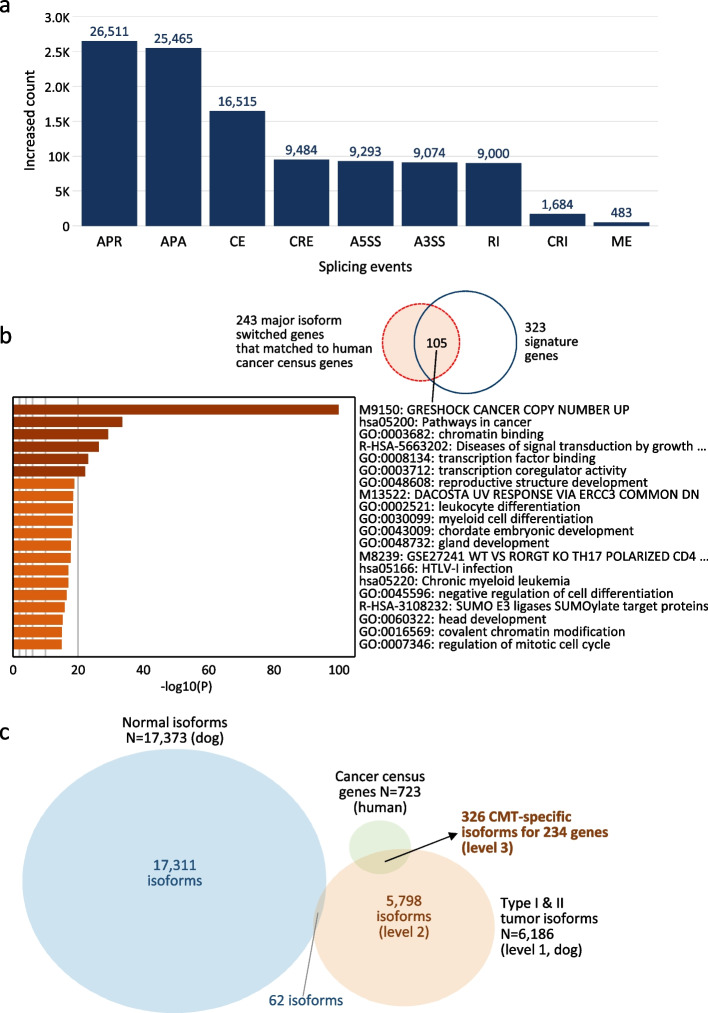
Isoform-centric analysis results. (**a**) Increased count of alternative splicing events in tumor condition compared to normal. Three events (APR, APA, and CE) showed the largest increase in the order. (**b**) Functional analysis results for 243 major isoforms switched genes that matched with human cancer census genes. Among them, 105 genes were enriched in a category related to cancer copy number alteration. (**c**) Venn diagram showing the selection procedure of tumor-specific isoforms. By excluding the overlapped normal isoforms and matching them to human cancer census genes, a total of 326 tumor isoforms (level 3) were selected as “CMT-specific isoforms

Abnormal splicing has been reported to be associated with the initiation and progression of human cancer [39]. Thus, it was investigated which alternative splicing events showed differences between normal and tumor conditions in dogs. Since more novel transcript isoforms were discovered in the tumor condition than in the normal one (Fig. 1b; Additional file 2: Table S2), all splicing events were increased in the tumor condition (Fig. 5a; Additional file 1: Fig. S7). Among them, APR and APA were the top 2 events with the largest increase, suggesting their contribution to mRNA-level diversity as reported in previous studies [40, 41]. And the third most event was CE, indicating differential exon usage in the corresponding genes between normal and cancer states, which is consistent with the results in human breast cancer cells [42]. For a gene of interest, users can easily find the changes in splicing patterns in tumor condition through the search or comparison functions implemented in the CanISO web page (Additional file 1: Fig. S8).

##### Major-isoform-switched genes during tumorigenesis

Major-isoform-switched genes were previously observed according to the condition change from normal to tumor, and it was suggested that they have potential as diagnostic biomarkers [22]. Accordingly, the number of cases across all genes was investigated in canine tumor samples. Among a total of 30,951 genes, 5,425 major-isoform-switched genes during tumorigenesis were found. Furthermore, to find a connection between the major-isoform-switched genes with cancers, we examined the number of cancer-related genes present among them by matching gene symbols to human cancer census genes (*N* = 723). We found that 243 of the major-isoform-switched genes were cancer-related genes, representing strong enrichment of cancer census genes (33.6%; 243 of 723; *P*-value = 3.7 × 10^–26^). Functional analysis of the matched genes revealed that 105 of them were enriched in a category related to copy number alteration in cancer (M9150: GRESHOCK CANCER COPY NUMBER UP; Fig. 5b; Additional file 2: Table S3), which was about one-half of the matched genes (105 of 243) and nearly one-third of the signature genes (105 of 323). These results demonstrate that the determination of major-isoform-switched genes could be an approach to identifying new cancer-related genes, a potential application of isoform-centric analysis.

##### CMT-specific isoforms

The discovery of tumor-specific isoforms holds promise for the diagnosis, prognosis, and therapy of human cancers [43, 44]. Their detection from extracellular (or secretory) RNAs would be a useful non-invasive tool for these purposes [45]. Such efforts in CMTs would facilitate the molecular marker development of human breast cancer. Thus, the identification of candidate RNA markers was attempted using transcript isoform expression and annotation. First, a total of 6,088 and 563 type I and II tumor isoforms were identified in the tumor condition (N_unique_ = 6,186; level 1; Fig. 5c), among which novel isoforms were most common (5,808 (94.7%) and 476 (84.4%), respectively). After the detection of 17,373 normal isoforms with sufficient expression levels (≥ 1.0 median TPM) and occupancies (≥ 10%) in the normal condition, 6,124 tumor isoforms (level 2) were obtained by filtering out 62 tumor isoforms that appeared to be detectable in the normal condition. Finally, 326 tumor isoforms were obtained that corresponded to 234 genes matched to the human cancer census genes (level 3; Fig. 5c). Since these isoforms originated from known cancer-associated genes and were observed only in the tumor condition, they were designated as “CMT-specific isoforms.” In addition, it was found that most of the CMT-specific isoforms (308 out of 326 (94.5%)) were novel isoforms, suggesting that these may be more appropriate for tumor diagnosis than the use of known isoforms. It was also confirmed that 44% of genes (103 out of 234) have somatic mutations that were reported previously [8], revealing the potential simultaneous detection of target RNA molecules and their sequence variants.

##### Sequence comparison of transcript isoforms between dog and human

Using the generated nucleotide sequences of transcript isoforms, we tried to examine how much overlap exists between the dog and human isoforms. For gene by gene comparison, we first identified 16,375 of 30,951 dog genes (52.9%) whose symbols are matched with those of human genes (N = 57,820) as shown in Additional file 1: Fig. S9. Therefore, we could compare 114,497 dog isoforms (38,394 known and 76,103 novel ones) with 118,852 human isoforms (92,459 known, 9,329 novel, and 17,064 putative ones).

From the sequence comparison results between dog and human isoforms (Additional file 1: Fig. S9), we observed 13,018 of 38,394 dog known isoforms (33.9%) having high similarity (≤ 0.1 distance) with human known ones, revealing approximately 1/3 overlap between the two isoform sets. Especially, we confirmed that more than 1/3 dog novel isoforms are almost matched with human known ones by observing 30,186 (39.7%) dog novel ones having high similarity with human known ones, which reveals that the discovery of novel transcript isoform in this study could contribute to expand current isoform-level annotation of dog transcriptome. In addition, 3,370 known isoforms (8.8%) and 4,616 novel ones (6.1%) in dog were only identified to have high similarity with human novel ones.

#### Transcriptome similarity between dog and human

In this study, we also provide gene-level expression data for dog tumor and even normal samples, which can be utilized to compare with those of other species to show transcriptome similarity. To this end, dog gene expression data in normal and tumor conditions were compared with 30 human tissues from GTEx project [30] and 32 human cancer types from NCI GDC Data Portal [32], respectively. In this comparison, we used median expression levels of the common genes (*N* = 16,375; Additional file 1: Fig. S9) in each tissue.

As expected, CMT samples were closest to human BRCA (spearman coefficient = 0.803; Additional file 1: Fig. S10a), which was consistent with the fact that CMT has been known as a great model for human BRCA [7, 8, 12]. Furthermore, dog normal mammary tissue was also closest to breast tissue in human (spearman coefficient = 0.839; Additional file 1: Fig. S10b). From these results, we could confirm transcriptome similarities in tumor and even normal conditions between dog and human. Therefore, we anticipated that dog gene expression data provided in this study can be utilized for comparison at the gene level narrowly or the whole transcriptome level broadly in other studies.

#### Identification of key signature pathways based on scores

Using read count matrices in normal and tumor conditions and KEGG pathway information [35], we determined scores of the pathways for CMT and human BRCA samples. To identify key signature pathways according to the condition change from normal to tumor, we sorted the pathways with cumulative dysregulation and differential regulation scores (RANK column) in descending order (Additional file 2: Table S4). In the results, we observed 7 out of the top 10 pathways were shared between both samples. Among them, the 1^st^-ranked pathway was “CELL CYCLE” as its importance is already known in tumorigenesis [46]. In the remaining pathways, we also identified crucial ones in cancer as following: FOXO SIGNALING PATHWAY [47], WNT SIGNALING PATHWAY [48], PI3K-AKT SIGNALING PATHWAY [49], and HIPPO SIGNALING PATHWAY [50]. Therefore, we demonstrated usefulness of score-based identification of key signature pathways that are altered during tumorigenesis, which may promote usability of CanISO database.

## Conclusions

In this study, we developed CanISO database containing genomic variations and transcriptome data by analyzing WES and RNA-seq datasets from large-scale dog samples. Web-based query systems were implemented on top of the database, not only allow detailed retrieval and display of canine genome and transcriptome data but also provide comparative analysis between different sample conditions or with human tissues. This study also contributes an expanded collection of transcript isoforms by uncovering novel isoforms in normal and tumor conditions. Furthermore, the applicability of the CanISO database was investigated through dog breed inference using SNP data, isoform-centric analyses using transcriptome data, transcriptomic similarity between dog and human, and score-based identification of key signature pathways. CanISO is projected to be a valuable genomic and transcriptomic data repository for researchers in related domains, as well as to expand the current understanding of these data, especially for cancer.

### Supplementary Information

**Additional file 1. **

**Additional file 2. **

## Data Availability

All the data are freely accessible for non-commercial academic use at https://www.kobic.re.kr/caniso/.

## Abbreviations

A3SS: 3' Splice sites
A5SS: Alternative 5' sites
APA: Alternative polyadenylation
APR: Alternative promoters
BAM: Binary Alignment/Map
BRCA: Breast cancer
CE: Cassette exons
CMT: Canine mammary tumor
CRE: Cryptic exons
CRI: Cryptic introns
GDC: Genome Data Commons
GTF: Gene transfer format
KEGG: Kyoto Encyclopedia of Genes and Genomes
ME: Mutually exclusive exons
NCI: National Cancer Institute
RI: Retained introns
RNA-seq: Whole transcriptome sequencing
TPM: Transcripts per million
VCF: Variant call format
VEP: Ensembl Variant Effect Predictor
WES: Whole exome sequencing
