# Supplementary material for: CanISO: a database of genomic and transcriptomic variations in domestic dog (Canis lupus familiaris)
Source: BMC Genomics. 2023 Oct 13;24:613. doi: 10.1186/s12864-023-09655-0 (PMC10571338; doi:10.1186/s12864-023-09655-0)
Supplement: Supplementary file 1 — Additional file 1. [file 12864_2023_9655_MOESM1_ESM.pptx]

## Slide 1
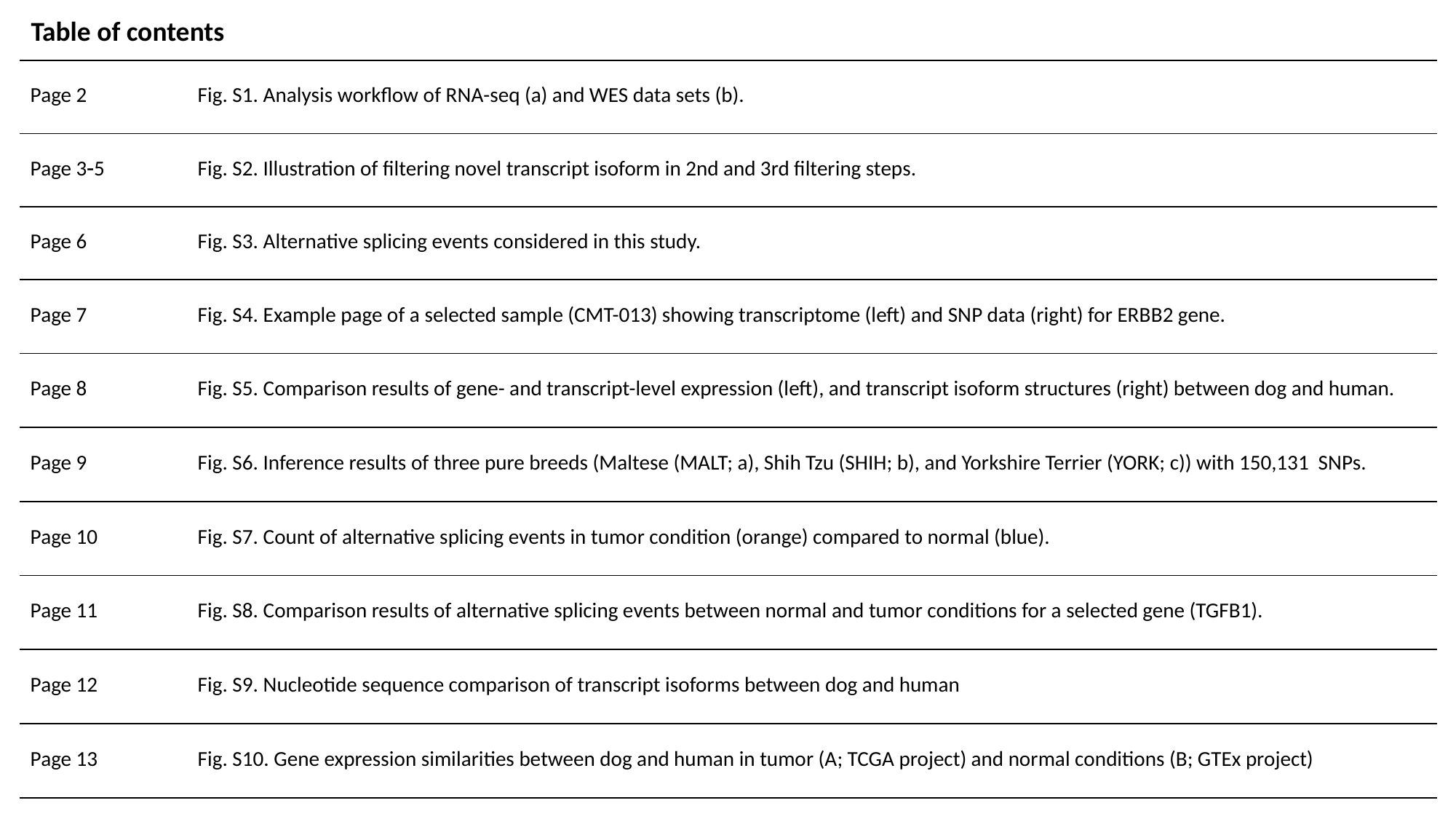

Table of contents
| Page 2 | Fig. S1. Analysis workflow of RNA-seq (a) and WES data sets (b). |
| --- | --- |
| Page 3−5 | Fig. S2. Illustration of filtering novel transcript isoform in 2nd and 3rd filtering steps. |
| Page 6 | Fig. S3. Alternative splicing events considered in this study. |
| Page 7 | Fig. S4. Example page of a selected sample (CMT-013) showing transcriptome (left) and SNP data (right) for ERBB2 gene. |
| Page 8 | Fig. S5. Comparison results of gene- and transcript-level expression (left), and transcript isoform structures (right) between dog and human. |
| Page 9 | Fig. S6. Inference results of three pure breeds (Maltese (MALT; a), Shih Tzu (SHIH; b), and Yorkshire Terrier (YORK; c)) with 150,131 SNPs. |
| Page 10 | Fig. S7. Count of alternative splicing events in tumor condition (orange) compared to normal (blue). |
| Page 11 | Fig. S8. Comparison results of alternative splicing events between normal and tumor conditions for a selected gene (TGFB1). |
| Page 12 | Fig. S9. Nucleotide sequence comparison of transcript isoforms between dog and human |
| Page 13 | Fig. S10. Gene expression similarities between dog and human in tumor (A; TCGA project) and normal conditions (B; GTEx project) |

## Slide 2
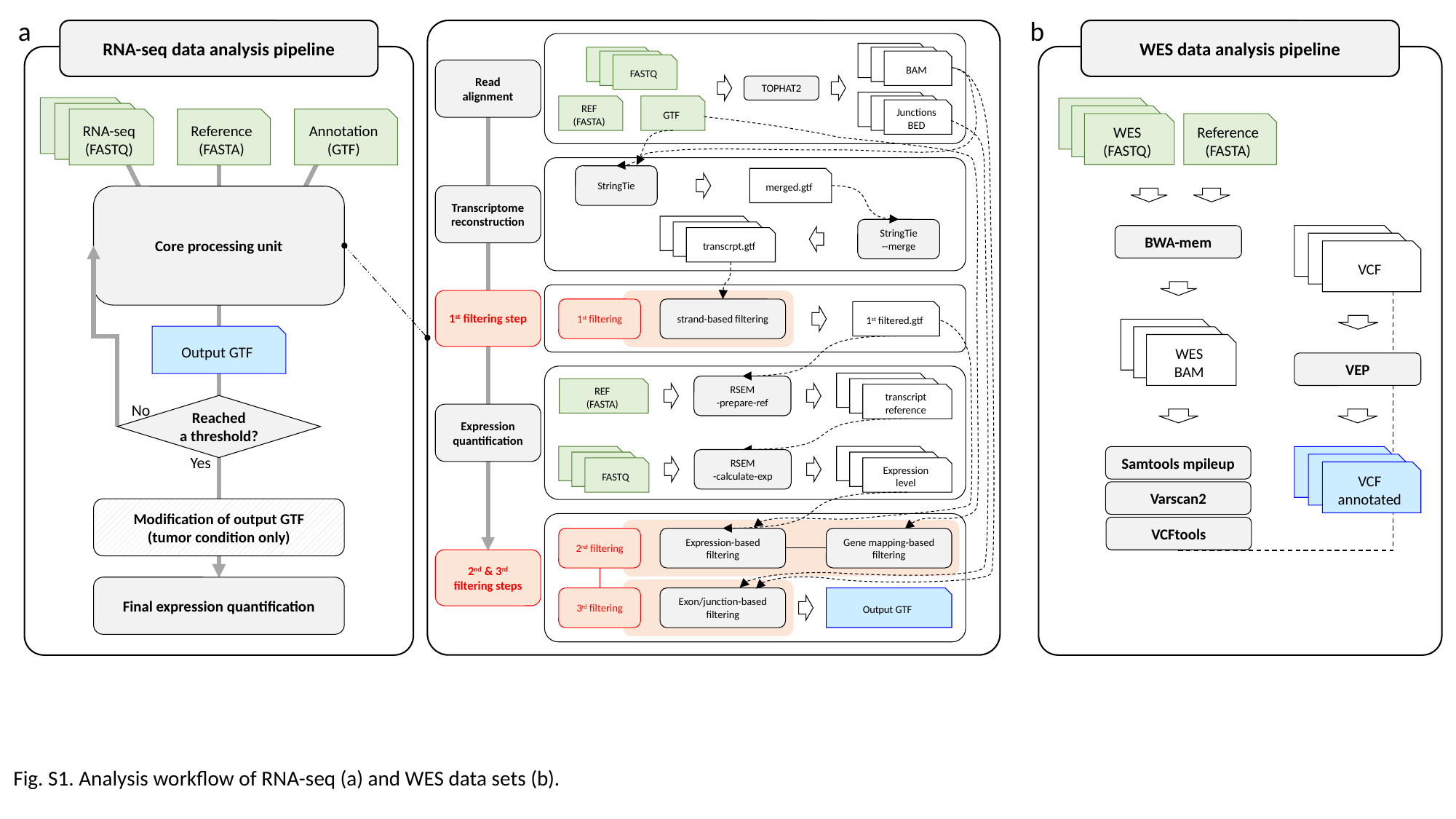

a
b
RNA-seq data analysis pipeline
RNA-seq
(FASTQ)
Reference
(FASTA)
Annotation
(GTF)
Core processing unit
Output GTF
No
Reached
a threshold?
Yes
Modification of output GTF
(tumor condition only)
Final expression quantification
BAM
FASTQ
TOPHAT2
Junctions
BED
REF
(FASTA)
GTF
StringTie
merged.gtf
transcrpt.gtf
StringTie
--merge
1st filtering
strand-based filtering
1st filtered.gtf
transcript reference
RSEM
-prepare-ref
REF
(FASTA)
FASTQ
Expression level
RSEM
-calculate-exp
2nd filtering
Expression-based filtering
Gene mapping-based filtering
3rd filtering
Exon/junction-based filtering
Output GTF
Read alignment
Transcriptome reconstruction
1st filtering step
Expression quantification
2nd & 3rd filtering steps
WES data analysis pipeline
WES
(FASTQ)
Reference
(FASTA)
VCF
VEP
VCF
annotated
BWA-mem
WES
BAM
Samtools mpileup
Varscan2
VCFtools
Fig. S1. Analysis workflow of RNA-seq (a) and WES data sets (b).

## Slide 3
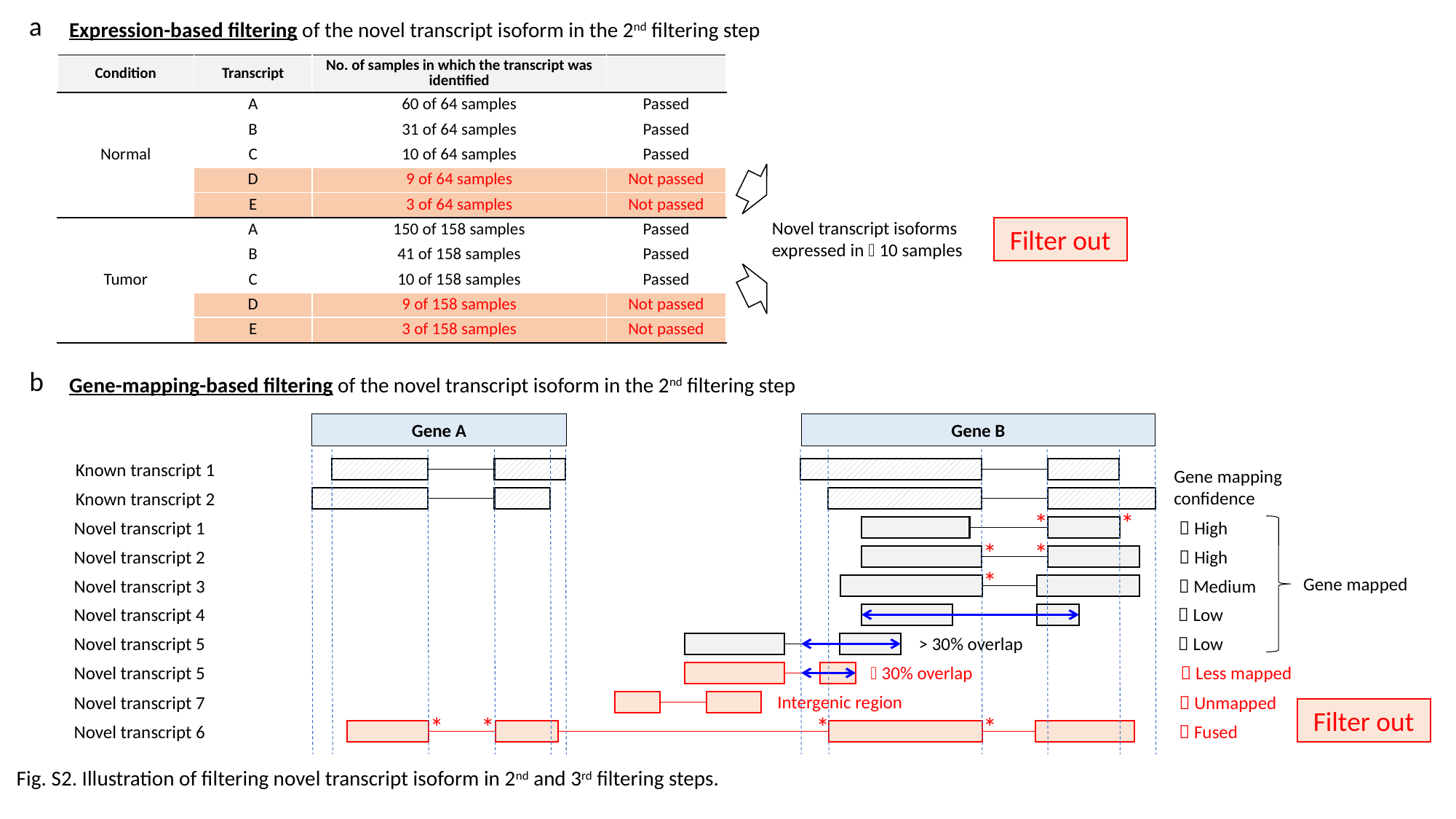

a
Expression-based filtering of the novel transcript isoform in the 2nd filtering step
| Condition | Transcript | No. of samples in which the transcript was identified | |
| --- | --- | --- | --- |
| Normal | A | 60 of 64 samples | Passed |
| | B | 31 of 64 samples | Passed |
| | C | 10 of 64 samples | Passed |
| | D | 9 of 64 samples | Not passed |
| | E | 3 of 64 samples | Not passed |
| Tumor | A | 150 of 158 samples | Passed |
| | B | 41 of 158 samples | Passed |
| | C | 10 of 158 samples | Passed |
| | D | 9 of 158 samples | Not passed |
| | E | 3 of 158 samples | Not passed |
Novel transcript isoforms expressed in  10 samples
Filter out
b
Gene-mapping-based filtering of the novel transcript isoform in the 2nd filtering step
Gene A
Gene B
Known transcript 1
Gene mapping confidence
Known transcript 2
*
*
 High
Novel transcript 1
*
*
 High
Novel transcript 2
*
Gene mapped
 Medium
Novel transcript 3
 Low
Novel transcript 4
> 30% overlap
 Low
Novel transcript 5
 30% overlap
 Less mapped
Novel transcript 5
Intergenic region
 Unmapped
Novel transcript 7
Filter out
*
*
*
*
 Fused
Novel transcript 6
Fig. S2. Illustration of filtering novel transcript isoform in 2nd and 3rd filtering steps.

## Slide 4
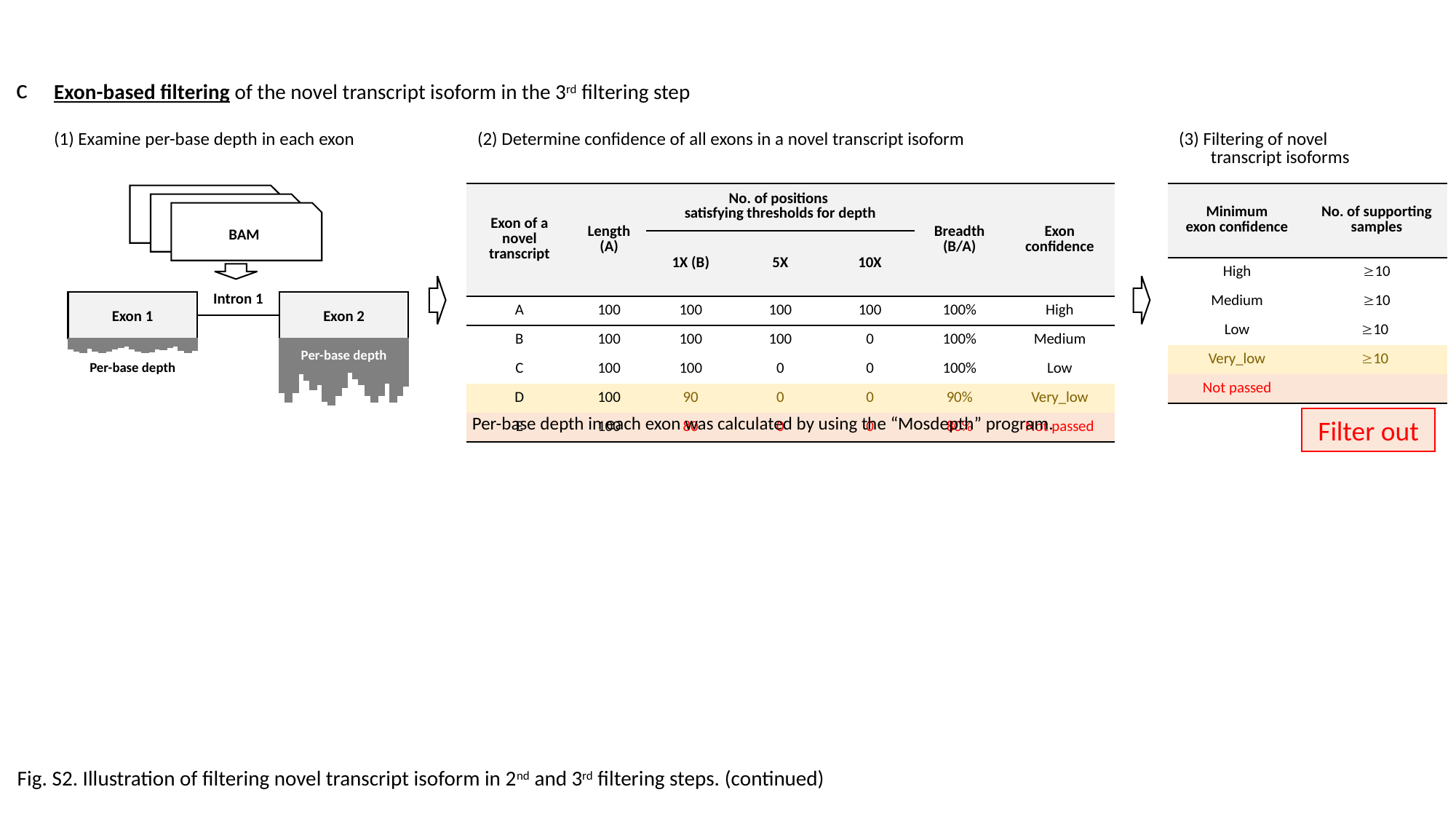

c
Exon-based filtering of the novel transcript isoform in the 3rd filtering step
(1) Examine per-base depth in each exon
(2) Determine confidence of all exons in a novel transcript isoform
(3) Filtering of novel transcript isoforms
| Minimum exon confidence | No. of supporting samples |
| --- | --- |
| High | ≥10 |
| Medium | ≥10 |
| Low | ≥10 |
| Very\_low | ≥10 |
| Not passed | |
| Exon of a novel transcript | Length (A) | No. of positions satisfying thresholds for depth | | | Breadth (B/A) | Exon confidence |
| --- | --- | --- | --- | --- | --- | --- |
| | | 1X (B) | 5X | 10X | | |
| A | 100 | 100 | 100 | 100 | 100% | High |
| B | 100 | 100 | 100 | 0 | 100% | Medium |
| C | 100 | 100 | 0 | 0 | 100% | Low |
| D | 100 | 90 | 0 | 0 | 90% | Very\_low |
| E | 100 | 80 | 0 | 0 | 80% | Not passed |
BAM
Intron 1
Exon 1
Exon 2
Per-base depth
Per-base depth
Per-base depth in each exon was calculated by using the “Mosdepth” program.
Filter out
Fig. S2. Illustration of filtering novel transcript isoform in 2nd and 3rd filtering steps. (continued)

## Slide 5
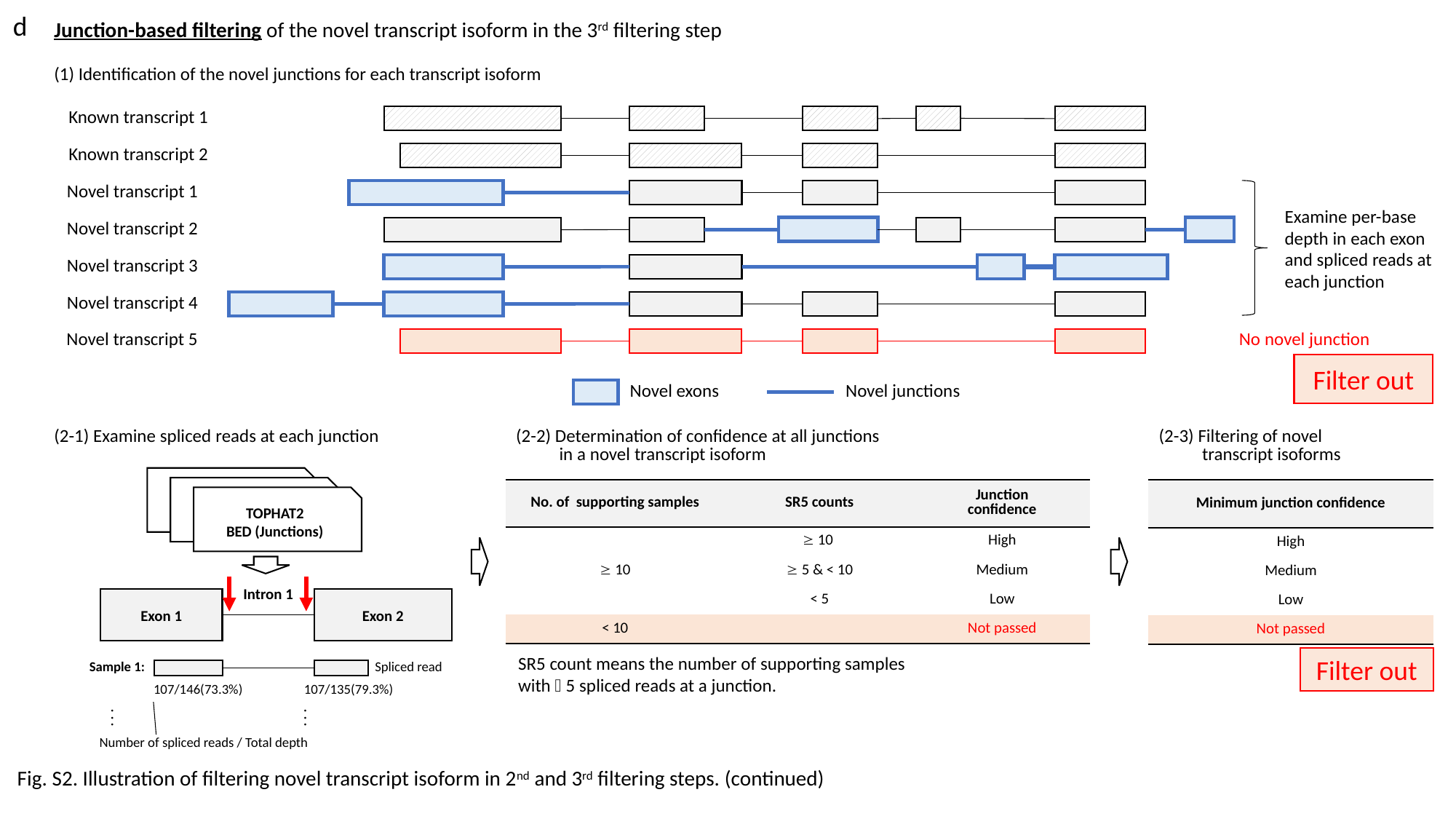

d
Junction-based filtering of the novel transcript isoform in the 3rd filtering step
(1) Identification of the novel junctions for each transcript isoform
Known transcript 1
Known transcript 2
Novel transcript 1
Examine per-base depth in each exon and spliced reads at each junction
Novel transcript 2
Novel transcript 3
Novel transcript 4
Novel transcript 5
No novel junction
Filter out
Novel exons
Novel junctions
(2-1) Examine spliced reads at each junction
(2-2) Determination of confidence at all junctions in a novel transcript isoform
(2-3) Filtering of novel transcript isoforms
TOPHAT2
BED (Junctions)
Intron 1
Exon 1
Exon 2
Sample 1:
Spliced read
107/146(73.3%)
107/135(79.3%)


Number of spliced reads / Total depth
| No. of supporting samples | SR5 counts | Junction confidence |
| --- | --- | --- |
| ≥ 10 | ≥ 10 | High |
| | ≥ 5 & < 10 | Medium |
| | < 5 | Low |
| < 10 | | Not passed |
| Minimum junction confidence |
| --- |
| High |
| Medium |
| Low |
| Not passed |
SR5 count means the number of supporting samples with ≥ 5 spliced reads at a junction.
Filter out
Fig. S2. Illustration of filtering novel transcript isoform in 2nd and 3rd filtering steps. (continued)

## Slide 6
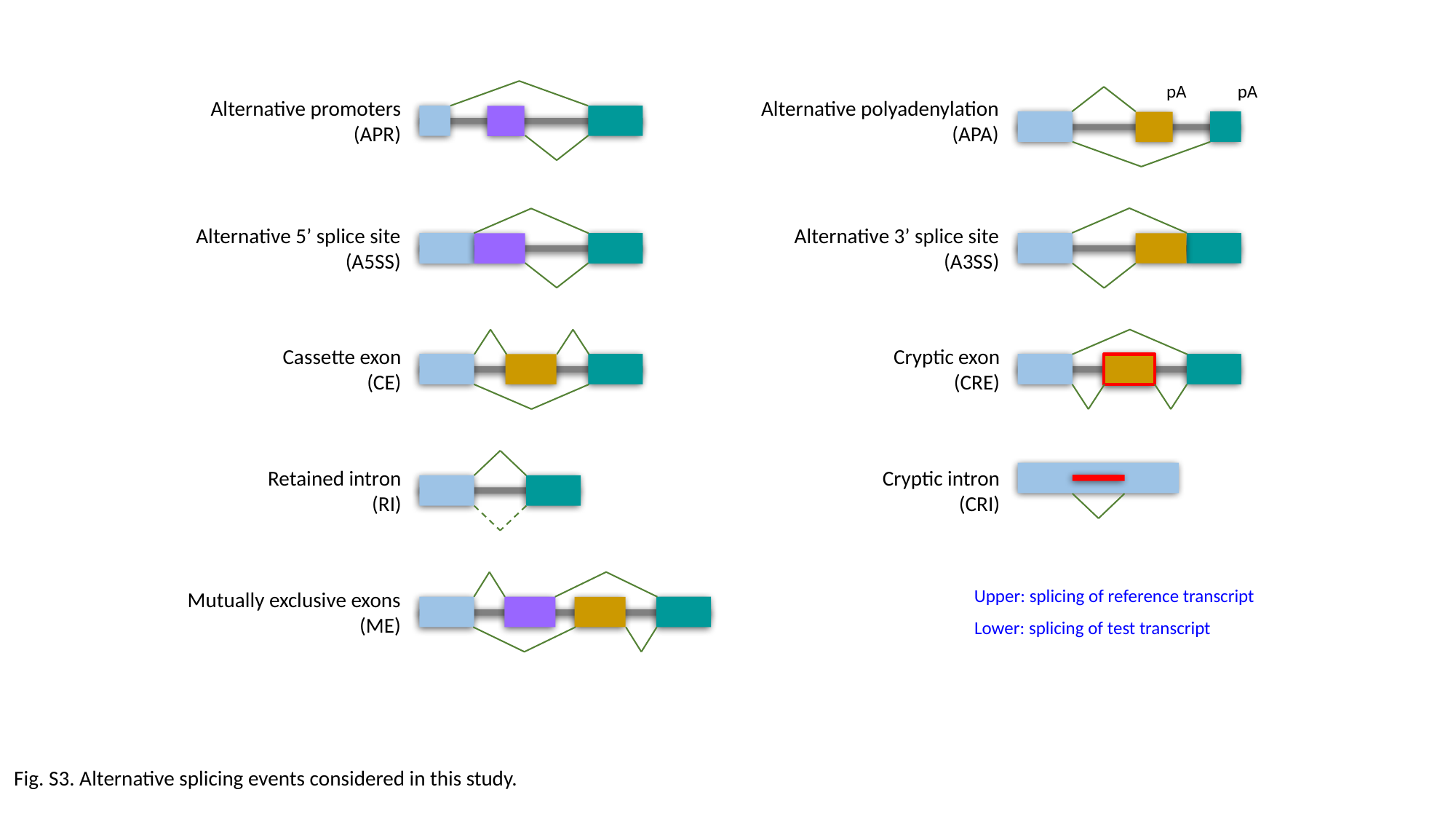

pA
pA
Alternative promoters
(APR)
Alternative polyadenylation
(APA)
Alternative 5’ splice site
(A5SS)
Alternative 3’ splice site
(A3SS)
Cassette exon
(CE)
Cryptic exon
(CRE)
Retained intron
(RI)
Cryptic intron
(CRI)
Upper: splicing of reference transcript
Mutually exclusive exons
(ME)
Lower: splicing of test transcript
Fig. S3. Alternative splicing events considered in this study.

## Slide 7
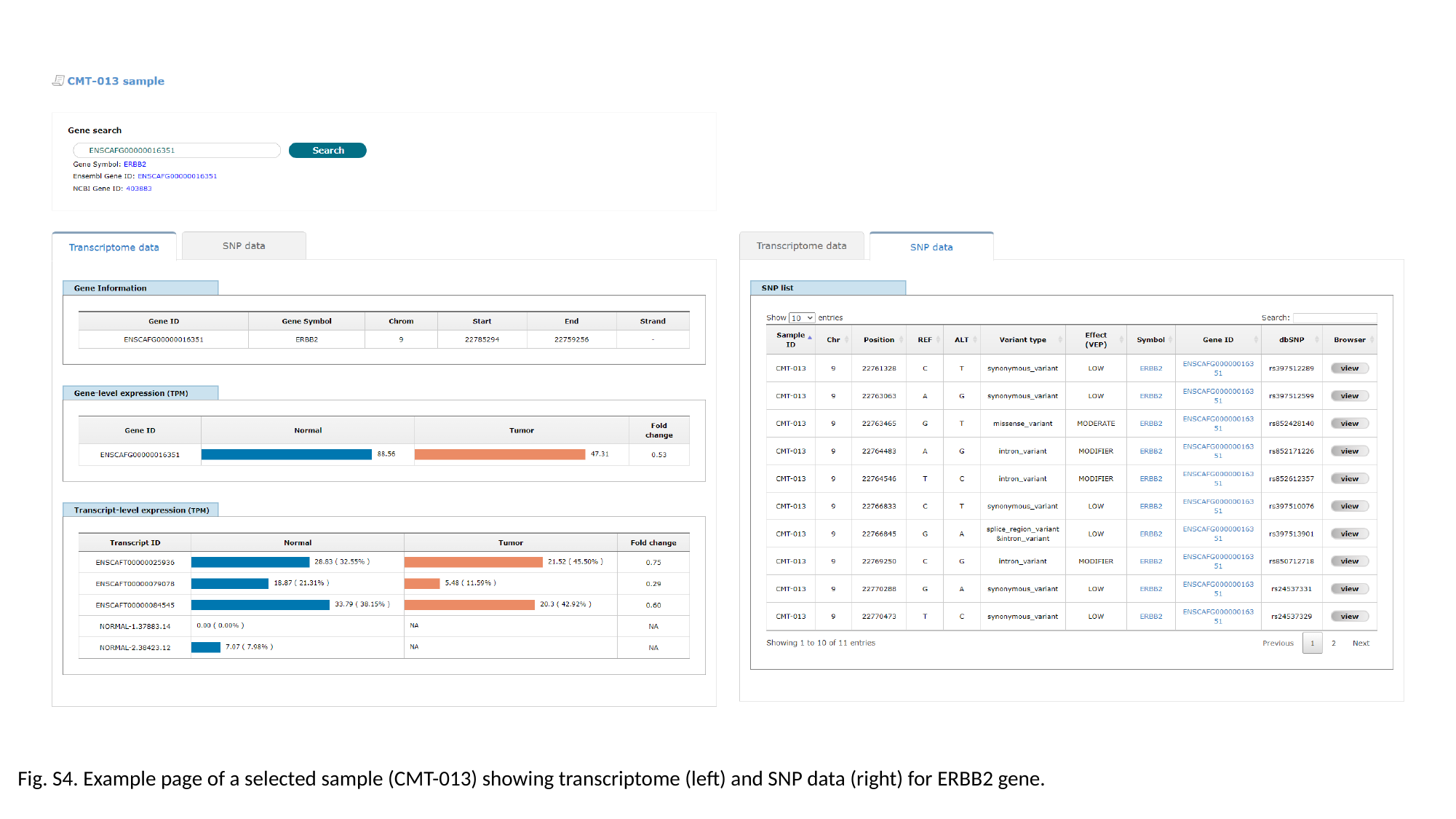

Fig. S4. Example page of a selected sample (CMT-013) showing transcriptome (left) and SNP data (right) for ERBB2 gene.

## Slide 8
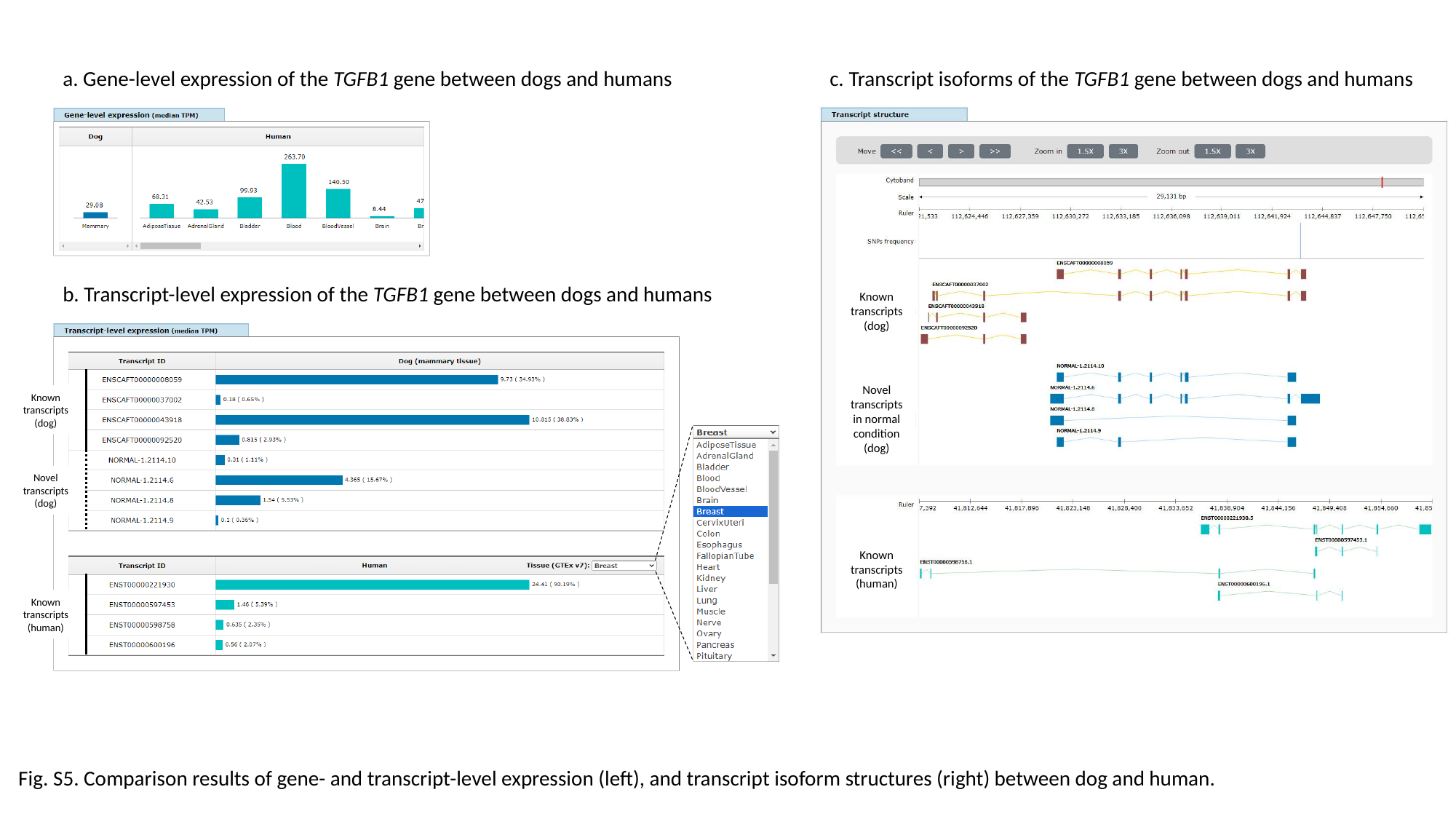

a. Gene-level expression of the TGFB1 gene between dogs and humans
c. Transcript isoforms of the TGFB1 gene between dogs and humans
Known transcripts
(dog)
Novel transcripts in normal condition
(dog)
Known transcripts (human)
b. Transcript-level expression of the TGFB1 gene between dogs and humans
Known transcripts (dog)
Novel transcripts (dog)
Known transcripts (human)
Fig. S5. Comparison results of gene- and transcript-level expression (left), and transcript isoform structures (right) between dog and human.

## Slide 9
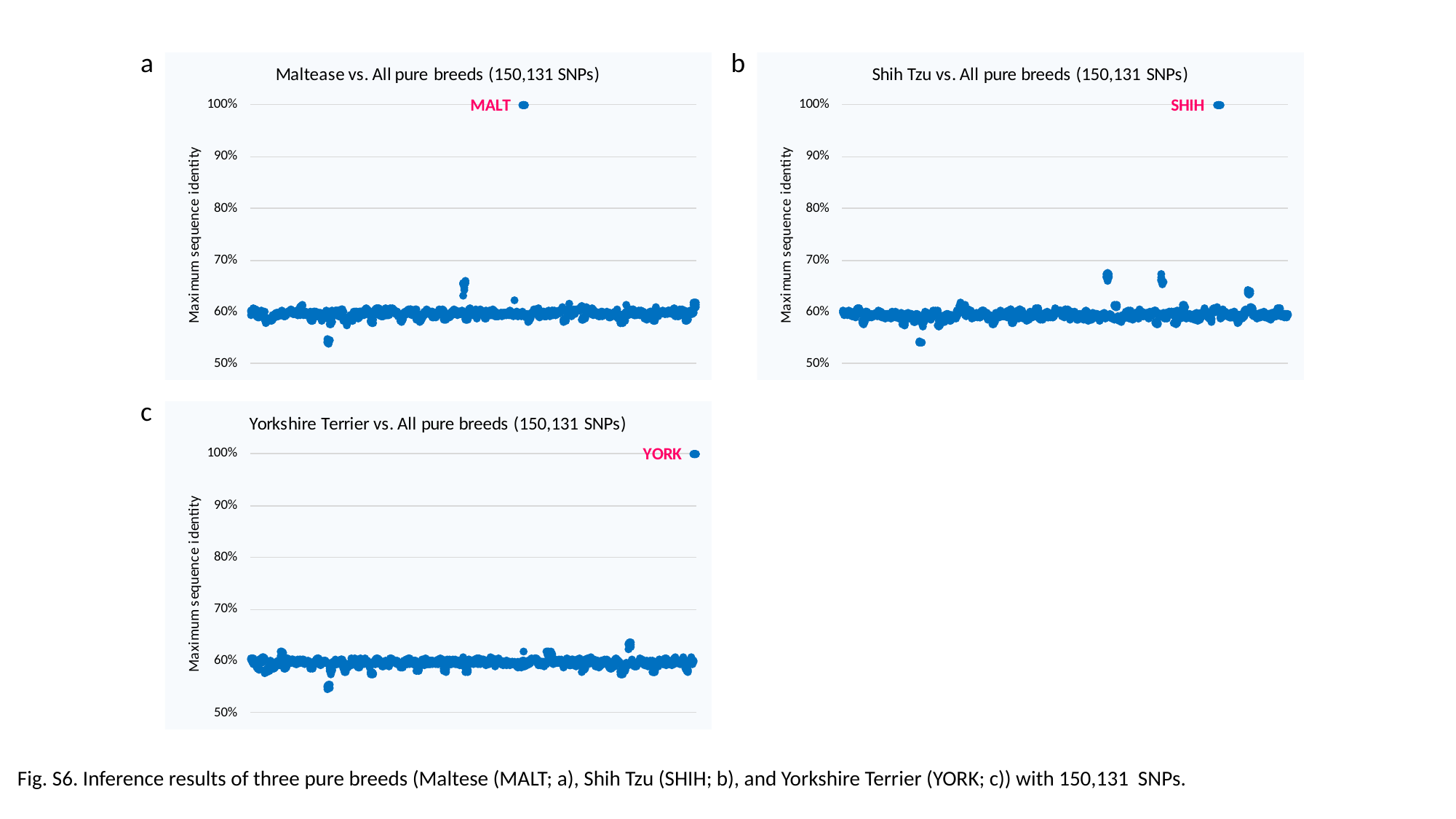

a
b
c
Fig. S6. Inference results of three pure breeds (Maltese (MALT; a), Shih Tzu (SHIH; b), and Yorkshire Terrier (YORK; c)) with 150,131 SNPs.

## Slide 10
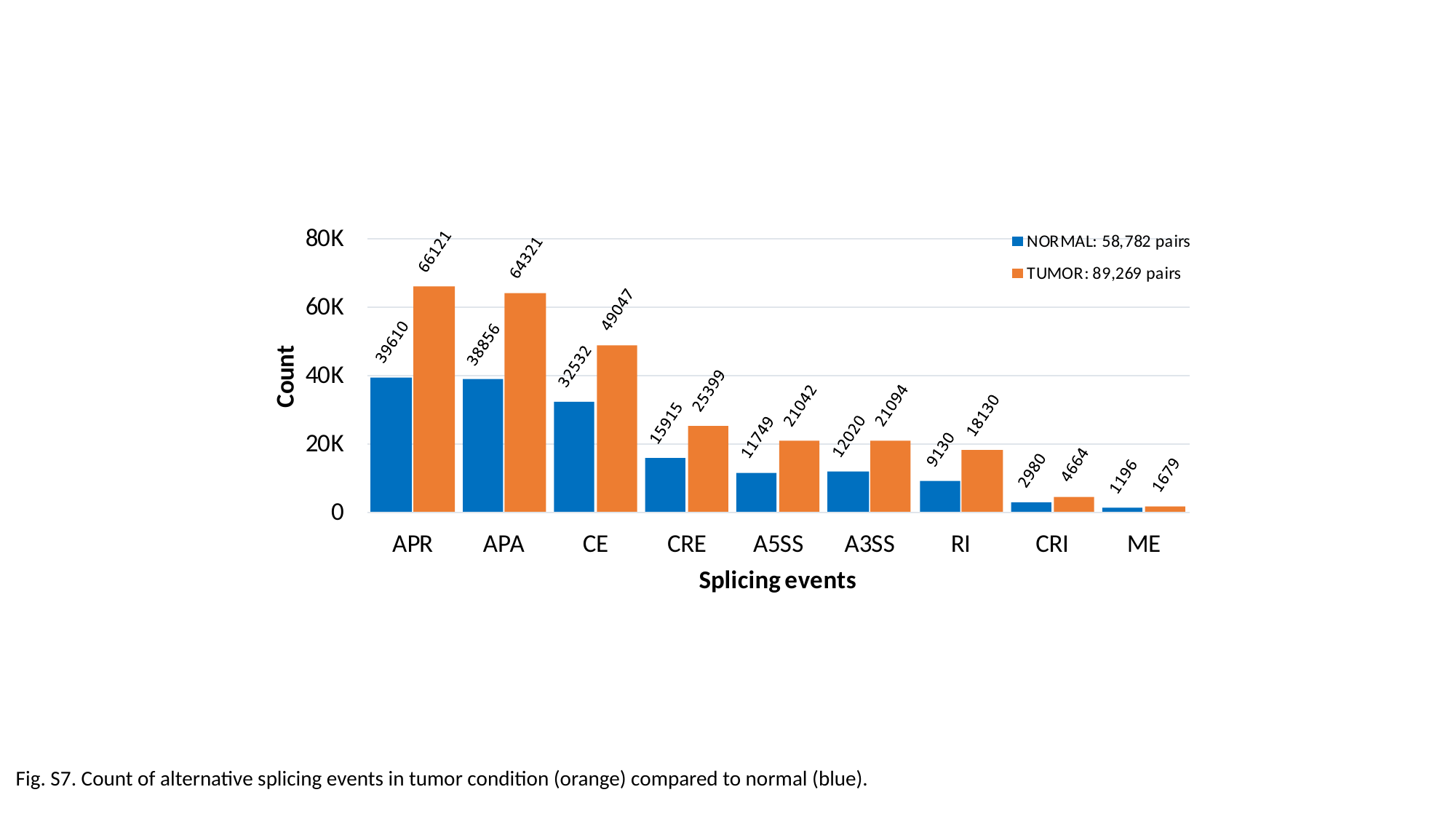

Fig. S7. Count of alternative splicing events in tumor condition (orange) compared to normal (blue).

## Slide 11
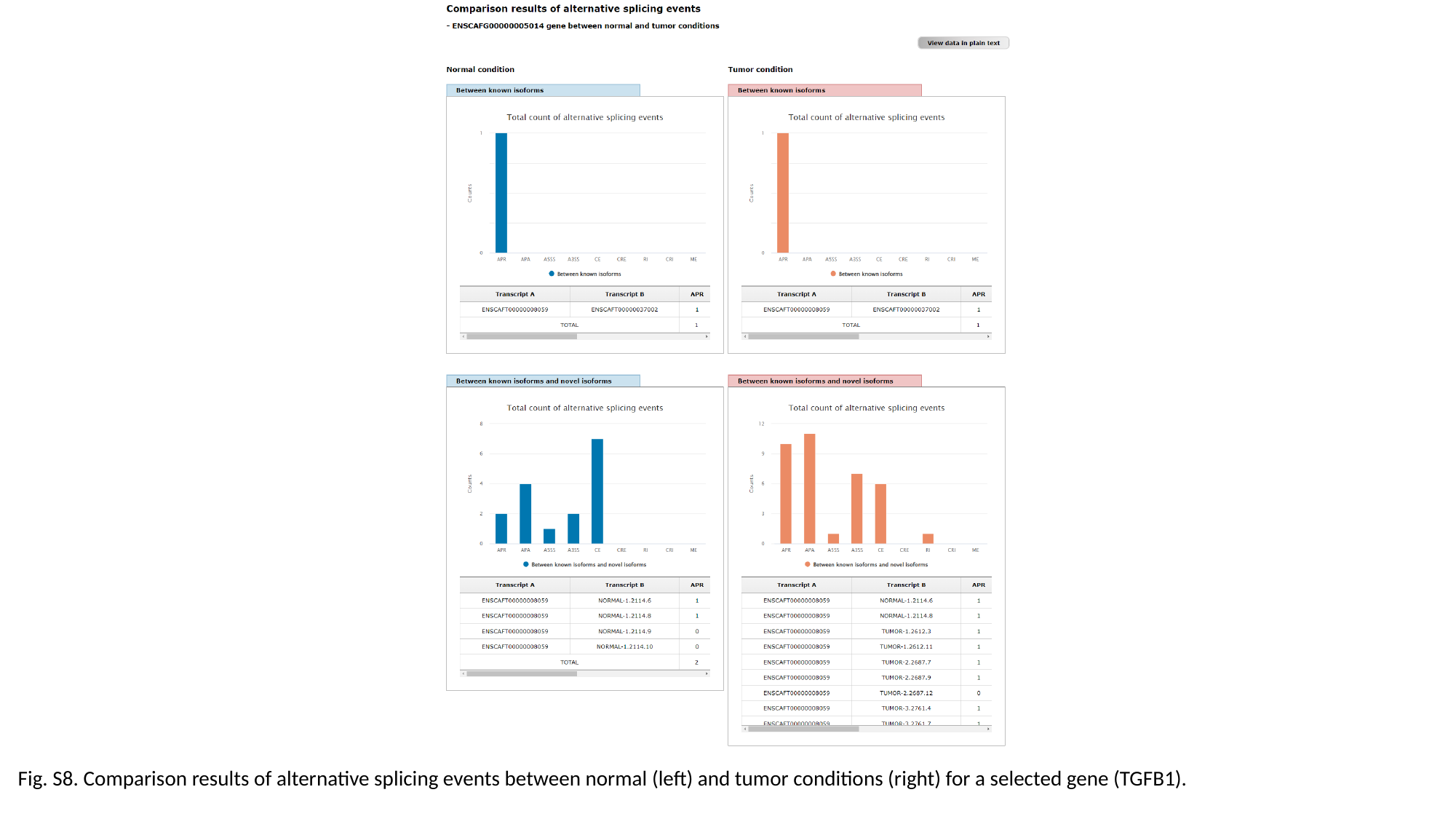

Fig. S8. Comparison results of alternative splicing events between normal (left) and tumor conditions (right) for a selected gene (TGFB1).

## Slide 12
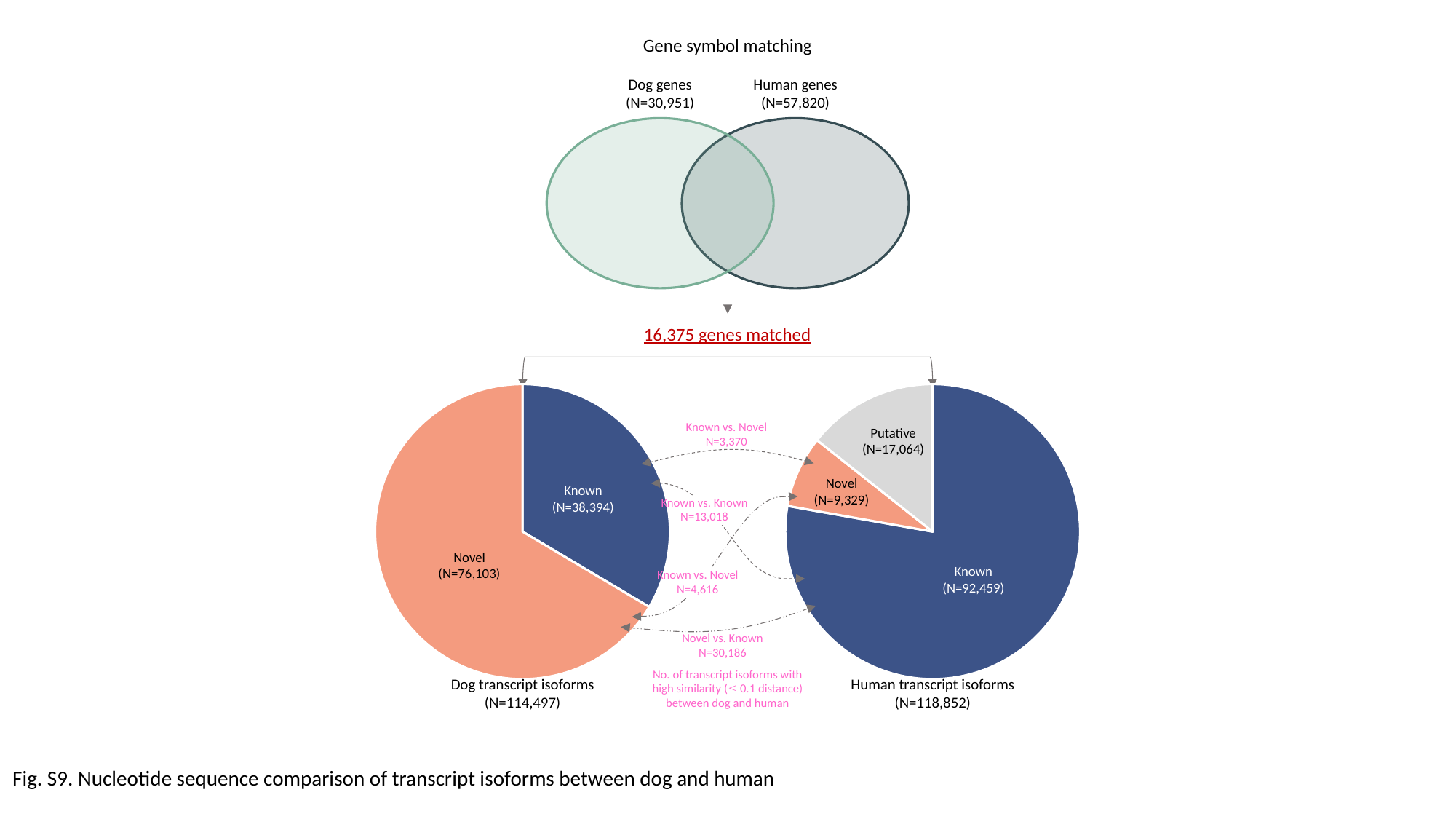

Gene symbol matching
Dog genes
(N=30,951)
Human genes
(N=57,820)
16,375 genes matched
### Chart
| Category | Dog |
|---|---|
| Known | 38394.0 |
| Novel | 76103.0 |
### Chart
| Category | Human |
|---|---|
| Known | 92459.0 |
| Novel | 9329.0 |
| Putative | 17064.0 |Known vs. Novel
N=3,370
Putative
(N=17,064)
Novel
(N=9,329)
Known
(N=38,394)
Known vs. Known
N=13,018
Novel
(N=76,103)
Known
(N=92,459)
Known vs. Novel
N=4,616
Novel vs. Known
N=30,186
No. of transcript isoforms with high similarity (≤ 0.1 distance) between dog and human
Dog transcript isoforms
(N=114,497)
Human transcript isoforms
(N=118,852)
Fig. S9. Nucleotide sequence comparison of transcript isoforms between dog and human

## Slide 13
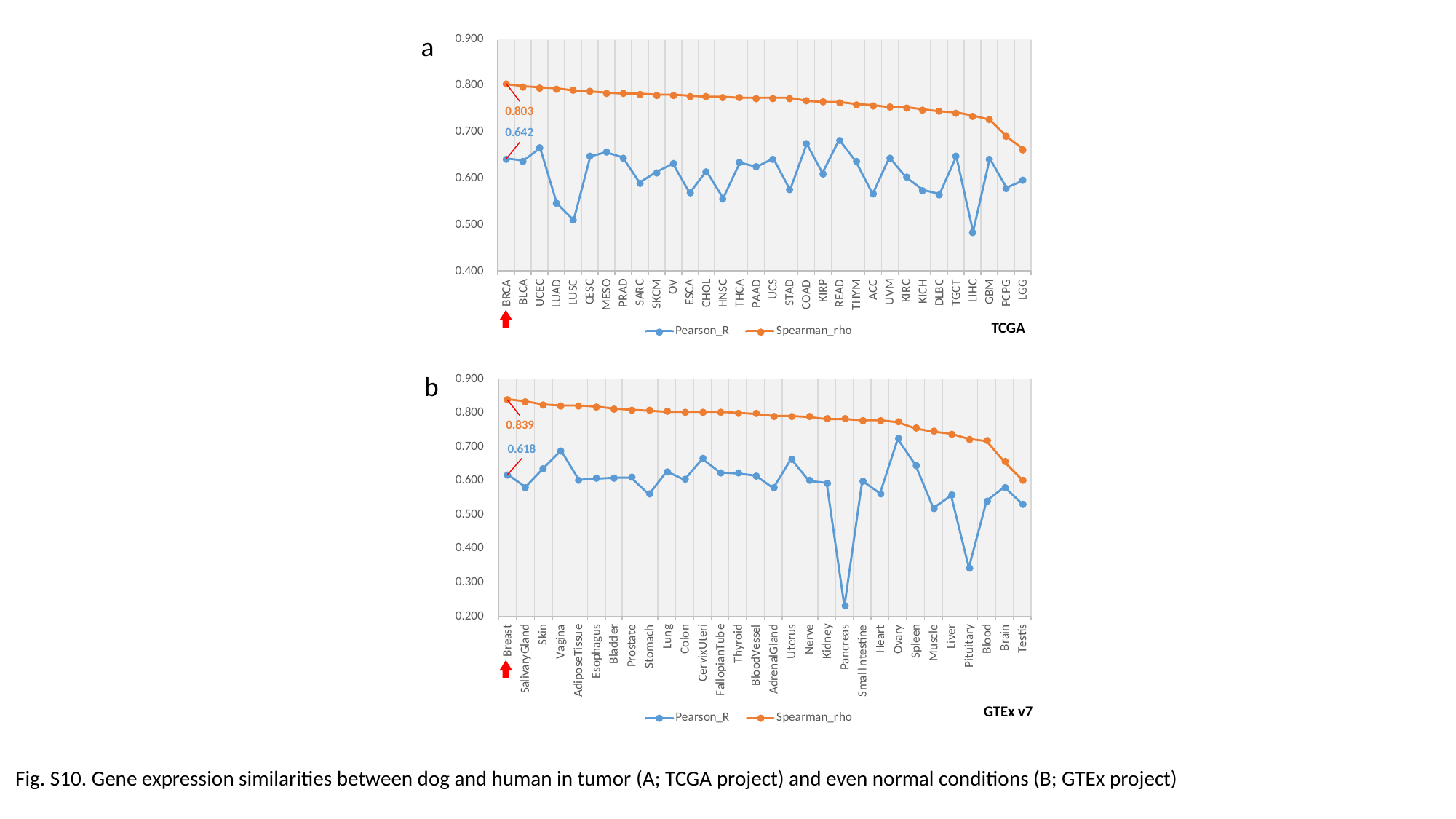

a
TCGA
b
GTEx v7
Fig. S10. Gene expression similarities between dog and human in tumor (A; TCGA project) and even normal conditions (B; GTEx project)
